# Inhibitors of the phosphodiesterase 2 increased axonal fibre growth in a dopaminergic organotypic *ex vivo* slice co-culture model

**DOI:** 10.1186/1471-2210-11-S1-P34

**Published:** 2011-08-01

**Authors:** Claudia Heine, Nico Scherf, Katja Sygnecka, Ute Egerland, Thorsten Hage, Heike Franke

**Affiliations:** 1 Translational Center for Regenerative Medicine (TRM), University of Leipzig, Leipzig, Germany; 2 Rudolf Boehm Institute of Pharmacology and Toxicology, University of Leipzig, Leipzig, Germany; 3 Biocrea, Radebeul, Germany

## Background

Due to the necessity for therapeutic strategies promoting neuro-(re)generation the development of appropriate models appraising the potential of substances for regeneration and repair of neuronal circuits are of great importance. Here we present a procedure for the quantification of fibre outgrowth on the basis of organotypic slice co-cultures of the dopaminergic projection system, appropriate for characterising the effects on axonal growth after treatment with well known controls and growth factors e.g. nerve growth factor (NGF) and new drug candidates. Therefore co-culture preparations were used, consisting of at least two slices of different parts of the brain, namely the ventral tegmental area/substantia nigra (VTA/SN) and the striatum (STR), whereas while incubation fibres projections regenerate and new connections were built, linking the two slices [1,2].

## Results

Utilizing immunohistochemistry in combination with laser scanning microscopy the outgrowing fibres in the border region of the VTA/SN-STR co-cultures were identified as microtubule associated protein-2 (MAP2)-, βIII-Tubulin- and tyrosine hydroxylase (TH)-positive. Furthermore the expression of phosphodiesterase (PDE) - 2A on cell bodies and fibres was revealed within the VTA/SN and STR. To expose the effect of the applied compounds on fibre outgrowth we developed a treatment protocol followed by tracing techniques and computerised quantification procedure. Thus we were able to quantify the fibre density together with detailed qualitative information about the growth characteristics of single fibres. Here we present recent data on stimulation studies using different phosphodiesterase (PDE) 2 inhibitors in comparison to NGF as well as control conditions. The incubation with the PDE2-inhibitor BAY 60-7550, ND 7001 and BTT5001 induced a significant increase of the fibre density in the border region of VTA/SN-STR co-cultures. The potential was comparable with the effect evoked by NGF. The involvement of PDE2 in fibre growth has been shown, suggesting an outgrowth promoting effect of PDE2 inhibitors. This is also supported by the elevation of intracellular cGMP upon inhibition of PDE2.

## Conslusion

The introduced *ex vivo* model of organotypic slice co-cultures provides a valuable tool to assess the therapeutic potential of drug candidates for regeneration and repair of disrupted neuronal circuits.
